# Supplementary material for: Mitochondrial ROS–ER Stress Axis Governs IL-10 Production in Neutrophils and Regulates Inflammation in Murine Chlamydia pneumoniae Lung Infection
Source: Cells. 2025 Sep 29;14(19):1523. doi: 10.3390/cells14191523 (PMC12524122; doi:10.3390/cells14191523)
Supplement: Supplementary file 1 [file cells-14-01523-s001.zip › Table S1.pdf]

Score	Cell infiltrate	Hemorrhage	Swelling of alveolar walls	Lung damage	Damage extension
0	Normal amounts of alveolar macrophages; no presence of leukocytes in alveoli or bronchioles.	No presence of erythrocytes in alveoli	Absent	Normal lung architecture	No lesions are observed
1	Moderate increase of leukocytes in alveoli and/or bronchioles	Limited presence of erythrocytes in alveoli	Moderate	Minimal change in lung architecture	Focal unilateral lesion
2	Severe increase of leukocytes in alveoli and/or bronchioles	Elevated presence of erythrocytes in alveoli	Severe	Moderate change in lung architecture with alveoli and bronchioles structures distinguishable	Focal and bilateral lesions/diffuse unilateral lesion
3	Cellular infiltrate does not allow to recognize bronchiole structure	—	Indistinguishable structure	Any lung structure can be recognized	Diffuse lesions in both lungs

Table S1. Histopathological score of lung tissue.
