# Supplementary material for: Mitochondrial ROS–ER Stress Axis Governs IL-10 Production in Neutrophils and Regulates Inflammation in Murine Chlamydia pneumoniae Lung Infection
Source: Cells. 2025 Sep 29;14(19):1523. doi: 10.3390/cells14191523 (PMC12524122; doi:10.3390/cells14191523)
Supplement: Supplementary file 1 [file cells-14-01523-s001.zip › Table S2.pdf]

	Forward	Reverse
Cxcl2	ACCCTGCCAAGGGTTGACTTC	GGCACATCAGGTACGATCCAG
Il1b	AAGGAGAACCAAGCAACGACAAAA	TGGGGAACTCTGCAGACTCAAACT
Il6	CCGGAGAGGAGACTTCACAG	CAGAATTGCCATTGCACAAC
Tnf	CCACCACGCTCTTCTGTCTAC	AGGGTCTGGGCCATAGAACT
Il4	GAACGAGGTCACAGGAGAA	TTTCAGTGATGTGGACTTGG
Il10	CAAAGGACCAGCTGGACAAC	CACTCTTCACCTGCTCCACT
Ifng	CTTGGATATCTGGAGGAAC	ATGAGCTCATTGAATGCTTG
Hspa5	TTCAGCCAATTATCAGCAAACTCT	TTTTCTGATGTATCCTCTTCACCAGT
Ddit3	CACATCCCAAAGCCCTCGCTCTC	TCATGCTTGGTGCAGGCTGACCAT
Eif2ak3	TCTGCCGACGATCAAATGGA	GGGCTGAGGATGGAAAAGCC
Eif2s1	ACGTGGCAGCCTTACACTAC	GGTCCGGCAAAGTTGGGATA
Atf4	GGGTTCTGTCTTCCACTCCA	AAGCAGCAGAGTCAGGCTTTC
Ern1	GAATTCCAATGCCGGCCCGGC	AAGCTTGGAGGGCGTCTGGAGTCAC
Atf6	GAGAGGTGTCTGTTTCGGGG	CAACTCCTCAGGAACGTGCT
Xbp1	GCAGCAAGTGGTGGATTTGG	CTGATGAGGTCCCCACTGAC
Actb	CACTGTCGAGTCGCGTCC	TCATCCATGGCGAACTGGTG
16S rRNA	GATTGCCAGTATAGATGCTTGTGAG	CTATGTCACTACTAACCCTTCCGCCACT

Table S2. Primer list
